# What COVID-19 has brought us to: Art, activism, and changes in social work education

**DOI:** 10.1177/1473325020973440

**Published:** 2021-03

**Authors:** Paula Gerstenblatt

**Affiliations:** University of Southern Maine, Maine, USA

**Keywords:** Art, story-telling, social work education, social justice, community work

## Abstract

This essay is a reflection about the COVID-19 pandemic from the vantage point of being on a sabbatical. As a result of the virus and global shut down, people are experiencing widespread suffering and economic devastation. The author, a professor, artist, and qualitative researcher advocates for a return to the profession’s community-based roots and an activist pedagogy. Additionally, as an artist/teacher/scholar, the author discusses the potential and importance of art and storytelling in social work education with examples of the author’s art created during the pandemic.

When COVID-19 hit I was one month into my sabbatical.

I had plans.

In addition to writing up my research, a case study of a veterinary clinic, I created a fantasy sabbatical of making art, travel, visits with friends, and rest. My first real break since graduating from my PhD program in 2013, I was looking forward to my sabbatical for over a year. I moved from my home in Portland, Maine to a 400 square foot cottage in Oakland CA behind a family home I co-own. I wanted to escape winter in Maine and spend time with family and friends in the Bay Area, where my Golden Retriever would be well cared for while I traveled. I set up a blog to chronicle my adventures, epiphanies, art, reflections on research, pedagogy, and life in general. I touched down January 31, 2020 in San Francisco, ready to roll.

**Figure 1.**
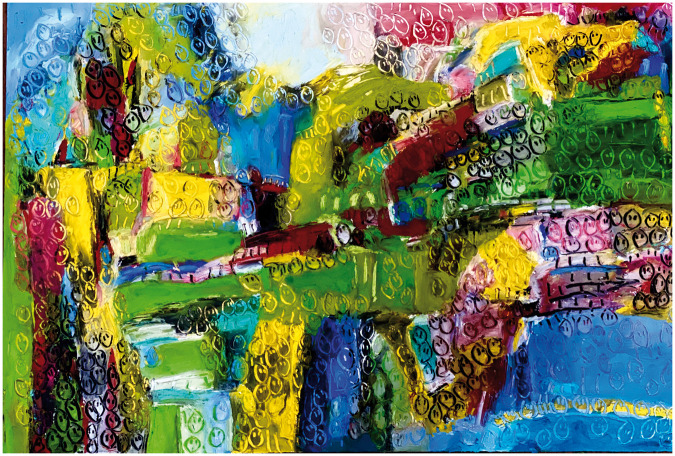
COVID-19 #4, Oil bar on paper.

I spent most of the first month adjusting to tiny living and setting up the cottage for writing and painting. Scaling down has challenges; however, this one bedroom cottage was designed well, and for the most part I had the room I needed and not an inch more. I started my blog in Portland, writing about my sabbatical fantasies, and more sadly, the death of my Golden Retriever, Pandy, after a three-year battle with cancer. My remaining dog, Pearl, was dazed and confused with the sudden changes; however, we moved closer to each other and began life as a duo.

**Figure 2.**
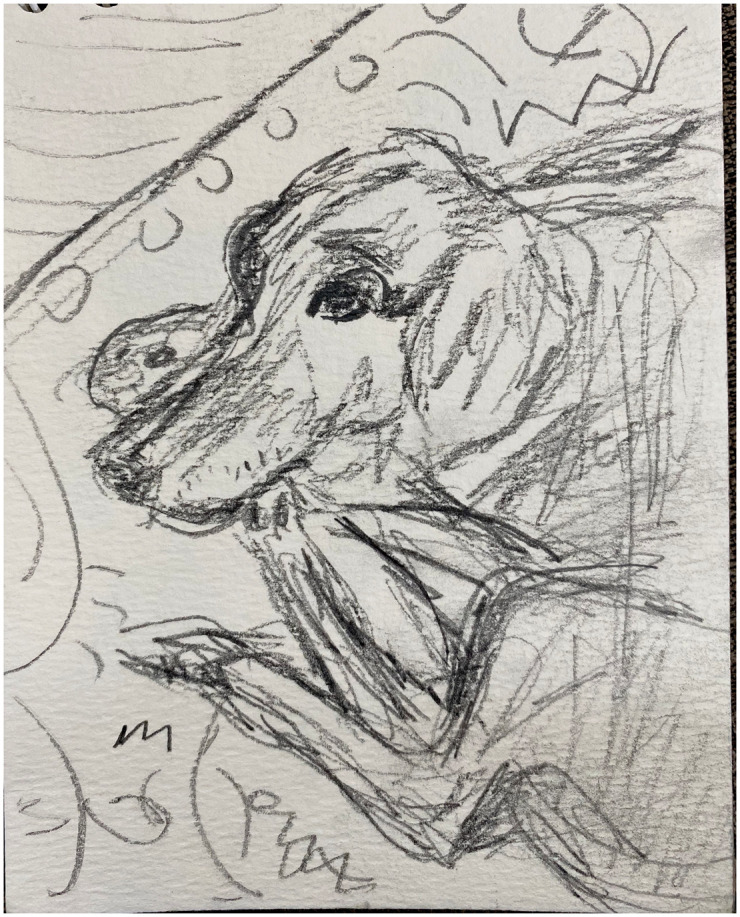
Pandy. Pencil sketch on paper.

I began a series of paintings about Oakland, starting with gentrification in West Oakland and then portraits of homeowners in our Oak Knoll neighborhood, which feels like a relic of the Oakland I used to love. Oak Knoll/Golf Links is a mixed neighborhood, with many Black homeowners living here for decades, as well as new Black families buying homes. Walking the hilly streets, I met neighbors and made new friends, many who agreed to be interviewed and photographed in front of their homes, share archival documents and old photographs for the collage paintings I promised to give them when I was done. I was excited with the art flowing, words filling pages of my blog, and warm sunny spring days with vibrant flowers blooming as snow fell in Maine. April and part of May were reserved for travel to Europe and road trips in the US. As February eased toward March, COVID-19 began to dominate the news and form a collective anxiety.

**Figure 3.**
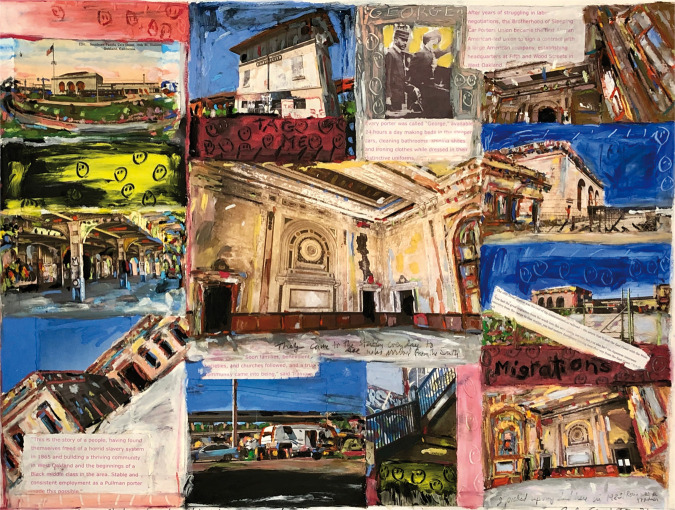
16th Street Train Station/West Oakland. Oil bar on paper.

The Bay Area was the first place in the US to issue a shelter in place order. We heard grumblings a few days prior and stocked up as best we could. It was as if a curtain was descending on our lives as I bid farewell to my sabbatical plans. I considered myself fortunate not to be making a rapid transition to online teaching, dealing with student anxiety, and endless Zoom meetings. My colleagues shared stories of the havoc and I found myself feeling gratitude with a dose of survivor’s guilt. Compared to those losing jobs, housing, and sitting in miles of cars lined up at food banks, I was one of the lucky ones with a paycheck and a roof over my head.

Yet, I became increasingly distracted and sad–mostly for others, but also for myself when I could admit it. My life resized and retracted. Sabbatical plans tumbling down a chute. Adventures canceled. Sleeping in a small room lined with paintings and a table covered with oil bars, the floor discolored from bits of paint ground into the wood with my sock. As my hand glided across the smooth white painting paper I thought *this is the map for my journey now.*

**Figure 4.**
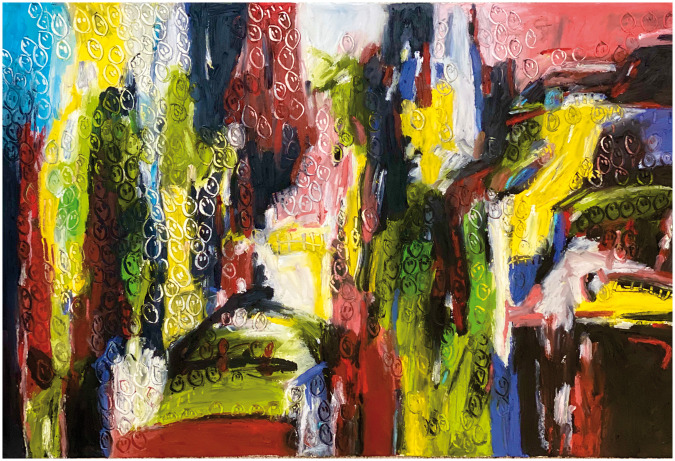
COVID#6. Oil bar on paper.

While I had a temporary reprieve from online teaching, it seemed certain the can was being kicked down the road. I never taught online and had no desire to. My teaching is project-based, a social justice service learning model informed by Freire’s *Pedagogy of the Oppressed* (Freire, 1971; Stoecker, 2003), The Learning Record (Styverson, 1995), a portfolio assessment centered on learning versus performance, and perhaps the most exciting is a multi-modal approach that includes visual art, mapping, music, poetry, and storytelling. I have written about the use of arts-based methods in research and practice, and recently added these approaches to my teaching (Gerstenblatt, 2013; Gerstenblatt et al., 2018).

In fall 2019 I ditched a textbook in the social justice/diversity MSW course and replaced it with six creative nonfiction and fiction books. In response to the assigned books students made a visual art piece about home, mapped a public space, created an annotated playlist, wrote two essays with prompts, and packed a small suitcase as was ordered by the government in 1942 when Japanese Americans were sent to internment camps. Students were terrified to work without point by point instructions; however, they surprised themselves and impressed me with their creative and compelling pieces. Using a multi modal approach allowed students to utilize all of their senses and learning styles. In a semester-long storytelling project, students interviewed a peer in the class; however, they were interviewed by a different student. The narratives were constructed using poetry, a quilt in the tradition of Faith Ringgold, maps, a story about an asylum seeker told through their cultural tradition of food, and visual narration using photos. Students may have grumbled at first, but most expressed gratitude for the readings, slight amazement at how much they learned, and tremendous pride in their projects.

Before COVID-19 I was looking for ways to upend aspects of my teaching, which prompted me to pass on the textbook and use multimodal assignments. I was an Osher Map Library Fellow in Spring 2019, and the amazing work my MSW policy students created from mapping a social problem confirmed my desire to increase creative assignments. In a K-12 feeder system saturated with high stakes testing, our students arrive at the university doorstep ready to chase the grade and get the degree. They are also terrified of making mistakes and experimenting. I spend a significant amount of time quelling anxiety, strengthening writing skills, and encouraging students to think. Based on Mezirow’s (1991) transformative learning theory, learning takes place when familiar frames of reference are removed. I try to minimize their anxiety, not their discomfort.

**Figure 5.**
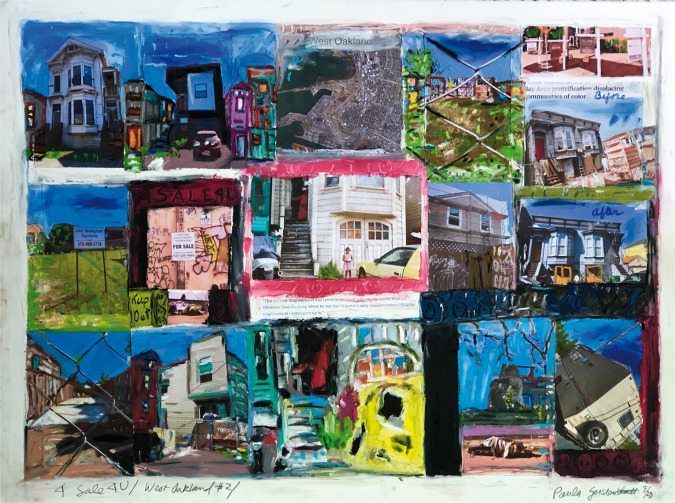
4 Sale 4 U/West Oakland #2. Mixed media collage on paper.

COVID-19 exposed our societal fault lines. Our professional values and practice are guided by the NASW Code of Ethics and social justice principles rooted in an activist community-based tradition led by iconic figures such as Jane Addams, Ida B Wells, Whitney Young, and Barbara Mikulski. The mass suffering has yet to be realized with over 40 million people out of work without health insurance, forthcoming evictions, lack of food, and the most vulnerable at risk of COVID-19 related health complications and death. This is particularly true for Black Americans, Latinx Americans, Native Americans, and poor people in general. As social work educators and qualitative researchers, we need to strengthen our pedagogy and research methods to elucidate the experiences and voice of people most often overlooked and hardest hit by COVID-19 and other structural failures of our social and economic system.

Symptoms of predatory capitalism and growing wealth inequality existed before COVID-19. Policies such as Medicare For All, $15 an hour minimum wage, student loan debt cancellation, de-incarceration, eliminating cash bail, legalizing marijuana, Green New Deal, wealth tax, paid parental leave, and universal child care were ginned up as radical, Socialist, and plagued by questions of how are you going to pay for it, even by those professing to be “liberal”. However; now we face a catastrophic economic collapse not seen since the Great Depression. We research these topics, teach them in class, and yet the activist tradition that defined our professional origins has been replaced with an emphasis on being clinicians, therapists, and mental health professionals.

While many see the professional divide as a false binary, including myself, the pendulum has nonetheless swung in a clinical direction. COVID-19 has put a chink in that armor, and beckons a return to our activist roots. We are compelled to rethink our pedagogy, research, and community work. It is fitting to ask ourselves who we are as social workers, teachers, scholars, and citizens as COVID-19 exposes and exacerbates the gross structural inequality and racism already in existence, reminding us we really haven’t been doing our job all along. Author Arundhati Roy frames the choice before us:

Historically, pandemics have forced humans to break with the past and imagine their world anew. This one is no different. It is a portal, a gateway between one world and the next. We can choose to walk through it, dragging the carcasses of our prejudice and hatred, our avarice, our data banks and dead ideas, our dead rivers and smoky skies behind us. Or we can walk through lightly, with little luggage, ready to imagine another world. And ready to fight for it. (Roy, 2020)

The need to fight for the reimagined world Roy eloquently describes requires a more activist pedagogy. Our department voted to offer classes online, either synchronous, asynchronous, or in combination. This feels daunting to someone who has never taught online and loves in person engagement with my students. I’ll miss lingering after class to discuss their community-based projects, listening to them excitedly talk about a reading, and helping them to revise assignmentns with heads bent over their paper.

Developing my Fall 2020 syllabi for online teaching, I intend to center classes on COVID-19. If we are studying FDR and the WPA, we need to look at how economic pump priming and government job creation could be advocated today, and come up with a plan to do just that. Studying the health care delivery system in the US compared with other industrialized nations has to be more than a policy comparison, it must include developing strategies to ensure those who have lost healthcare in an employer-based system are not left high and dry. That would mean advocating for Medicare For All by putting pressure on politicians, running for office, and involving those most impacted. Activism takes many forms, whether we are on the frontlines, formulating policy, developing new online pedagogy, organizing relief efforts in our communities, or the tellers of stories, which qualitative researchers are well positioned to do.

Being on sabbatical, I was able to reflect without the pressure of the day to day grind. I have been thinking about how COVID-19 has changed me and how to use this change for the better. Daily peaks and valleys find me bursting with joy as I paint or dive into research, only to slide into the COVID blues and ask myself what’s the point? Who really cares about this blog post or painting? How will this reading or assignment be meaningful to my students, particularly for those trying to cope with loss, financial pressure, balancing work, school, and homeschooling children at the same time? What is the best way for me to contribute? For me the answer to this question has always been the same–tell the stories.

**Figure 6.**
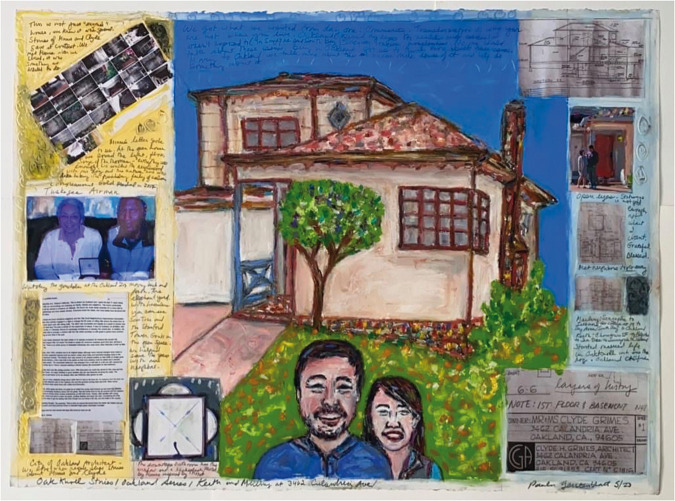
Oak Knoll Stories/Oakland Series/Keith and Meiling at 3462 Calandria. Collage on paper.

An artist friend and I were discussing making art in the time of COVID. Is it self-indulgent or insensitive to savor this unexpected bounty of time to create? Perhaps; however, I believe it is my job as an artist/scholar to document stories in the best and worst of times. We are currently living an unfolding historic trauma and someday our art will become an important part of the collective narrative–what we saw, felt, lost, and gained. A smile forming while watching birds in flight. Finding a handwritten letter in the mailbox. Dancing at a live concert on a neighbor’s lawn. Allowing space for sadness, despair, anger. Noticing what we were too busy to see or feel. Details of our confinement spaces–the green kitchen sink, art table, pile of books, trees outside the window, cityscapes and landscapes. Across a chasm, street, or country. The lines of cars at food banks, bodies in cold storage, unrelenting grief, voices heard and overlooked. What saved or nearly sunk us.

**Figure 7.**
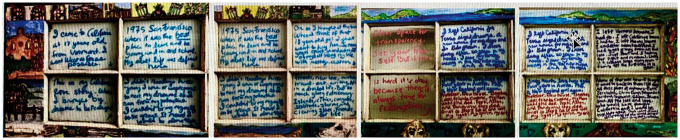
My California Love Story. Oil pastel on reclaimed French door.

When the COVID blues grip my soul, I’ll remember blues are part of the telling. And when I struggle to answer the what’s the point question–why paint, write, teach, or yearn for a future I can no longer imagine, it will be to survive and pay tribute to those who fell. We need a better world that is more than gliding over one false bottom after the other. My job is the telling–as an artist, scholar, teacher, and human. At our best we are dispensers of truth, and hope, for without it there is no other side to fight for.
